# Serous retinal detachment and multiple retinal pigment epithelial detachments, following hemodialysis for multi-organ failure

**DOI:** 10.4103/0301-4738.62670

**Published:** 2010

**Authors:** Soumyava Basu, Taraprasad Das, Tapas Ranjan Padhi

**Affiliations:** LV Prasad Eye Institute, Bhubaneswar, Orissa, India

**Figure 1:**
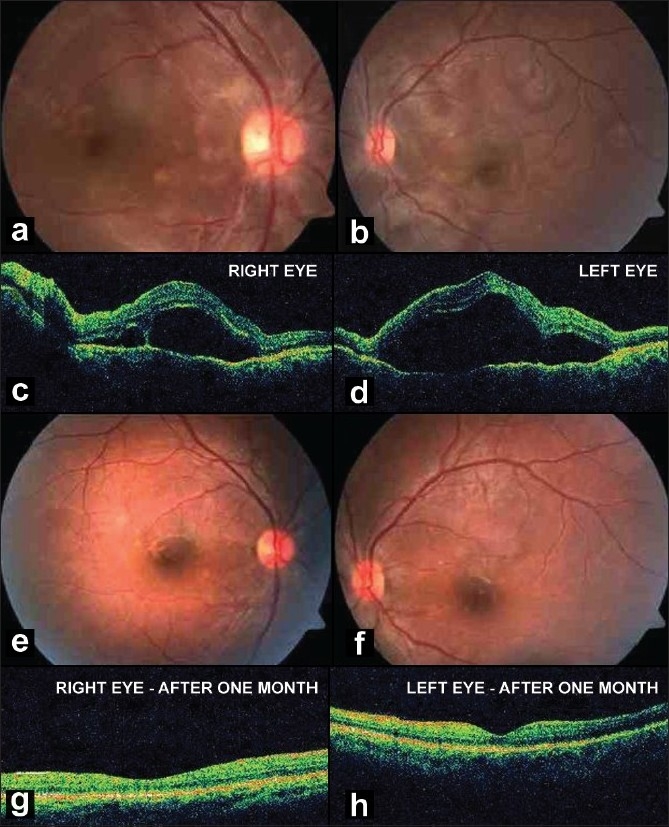
**(a-b):** Fundus photograph of both eyes showing multiple serous retinal detachments with pigment epithelial detachments. **(c-d):** OCT of both eyes at presentation, showing hypo-reflective area beneath the retina, suggestive of sub-retinal fluid. The line scan did not cross through any of the PEDs. **(e-f):** Fundus photograph of both eyes after one month follow-up, showing total resolution of sub-retinal fluid and PEDs, and residual pigment epithelial changes. **(g-h):** OCT after one month follow-up showing resolution of sub-retinal fluid with normal foveal contour

Dear Editor,

A 28-year-old male patient developed multi-organ failure, including acute renal failure, adult respiratory distress syndrome (ARDS) and acute pancreatitis following an attack of Falciparum malaria, for which he received renal replacement therapy with intermittent hemodialysis. After four cycles of hemodialysis, his uremic status improved partially (60 mg/dl), but he developed blurring of vision in both eyes, right more than left. On examination, his best corrected visual acuity (BCVA) was found to be 20/400 and 20/60 in the right and left eye respectively. External examination was normal in both eyes. Fundus examination revealed multiple pigment epithelial detachments (PEDs) over the posterior pole, with overlying multifocal serous retinal detachment (RD) in both eyes [Fig. 1a, 1b], which was confirmed with optical coherence tomography (OCT) [Fig. 1c, 1d]. Fundus fluorescein angiography was deferred in view of the patient's renal status. The patient was advised to continue his treatment for renal failure, and to review with us after one month. He received five more sessions of hemodialysis over the next two weeks, till his uremic status became normal. On follow-up (one month), his BCVA had improved to 20/25 in both eyes. Both the PEDs and the overlying sub-retinal fluid had resolved in both eyes (confirmed on OCT), leaving behind a few areas of retinal pigment epithelial atrophy [Fig. 1e, 1h].

The occurrence of PEDs with serous RD following hemodialysis has been reported previously.[1,2] It has also been reported after acute renal failure in systemic lupus erythematosus (SLE), in the absence of hemodialysis.[3] The mechanism for this phenomenon has however been debated. Gass[1] argued that renal failure and subsequent uremia is the primary mechanism for the formation of PED and subsequent serous RD. He noticed focal dehiscence in the pigment epithelium, at the margins of some PEDs. Troriano *et al.*[2] hypothesized that dialysis-induced hemodynamic shifts between various compartments, lead to altered permeability, and formation of PEDs. Intermittent hemodialysis has been shown to induce significant hypotension and hemodynamic intolerance in critically ill patients.[4]

Our patient developed visual symptoms, while he still had significant uremia (60 mg/dl). Besides, his visual symptoms improved, even though he received five more sessions of hemodialysis, after initial evaluation at our clinic. Both point to the possibility that the hemodynamic changes induced by intermittent hemodialysis or the dialysate used in it probably did not have a role in the ocular manifestations. Instead the presence of uremia and the changes in osmolarity, induced by it, in the intra-vascular and extra-vascular compartments could have a role in the movement of fluid into the sub-pigment epithelial and subsequently into the sub-retinal space. A similar condition, described in patients affected by nephritis due to SLE, who never underwent hemodialysis, also points to renal failure, as the possible culprit.[5]

As our patient had multi-organ failure, the role of acute pancreatitis and ARDS also needs to be probed. To the best of our knowledge, this is the first report of PED and serous RD in a case of multi-organ failure. It also highlights the possibility of spontaneous resolution in this condition. Laser photocoagulation, as mentioned in the previous reports,[1] is probably not required.
